# Extraterritorial hunting expeditions to intense fire scars by feral cats

**DOI:** 10.1038/srep22559

**Published:** 2016-03-02

**Authors:** Hugh W. McGregor, Sarah Legge, Menna E. Jones, Christopher N. Johnson

**Affiliations:** 1 Australian Wildlife Conservancy, Mornington Wildlife Sanctuary, PMB 925, Derby, WA 6728, Australia; 2 School of Biological Sciences, University of Tasmania, Private Bag 55, Hobart, Tasmania 7001, Australia

## Abstract

Feral cats are normally territorial in Australia’s tropical savannahs, and hunt intensively with home-ranges only two to three kilometres across. Here we report that they also undertake expeditions of up to 12.5 km from their home ranges to hunt for short periods over recently burned areas. Cats are especially likely to travel to areas burned at high intensity, probably in response to vulnerability of prey soon after such fires. The movements of journeying cats are highly directed to specific destinations. We argue that the effect of this behaviour is to increase the aggregate impact of cats on vulnerable prey. This has profound implications for conservation, considering the ubiquity of feral cats and global trends of intensified fire regimes.

Mammalian predators typically show high site fidelity, and many occupy territories from which other members of the same species are excluded, either by aggression or mutual avoidance^1^,^2^. Territoriality by predators is an important factor that allows stable coexistence of predators and prey^3^. Fidelity to their territories means that predators cannot rapidly change locations to track short-term shifts in distribution of prey^4^, and this can limit their total impact on prey populations. African lions *Panthera leo*, for example, occupy stable territories year-round, even in situations where their most abundant prey undergo seasonal migrations and so become inaccessible for part of the year^5^. Territoriality also dampens growth of predator populations, contributing to the lagged response of predator to prey population growth and allowing fluctuating prey populations time to recover from low density in systems with linked predator and prey population dynamics^3^,^6^,^7^,^8^. Without site fidelity by predators, therefore, predation rates averaged across space and over time can be higher^9^.

Long-distance extraterritorial movement by terrestrial mammalian predators is energetically expensive and physiologically stressful^10^,^11^, and exposes journeying animals to the risk of aggressive encounters with conspecifics^12^. Most documented long-distance movements of such predators involve juvenile dispersal^13^,^14^, individuals experiencing dire food shortages^15^,^16^, or males embarking on searches for mates^17^. However, there have been numerous observations of mammalian predators making extra-territorial movements seemingly in search of prey^18^,^19^, such as foxes *Vulpes vulpes* travelling to townships^20^ and hyenas travelling to elephant carcasses^21^. It is possible that many other mammalian predators make long-distance expeditions to localised pulses of prey availability; however, there is little documented evidence for this.

Populations of most species of small mammals are currently collapsing in Australia’s northern savannahs^22^. There is evidence that intensified fire regimes and predation by feral cats *Felis catus* are at least partly responsible for these declines^23^,^24^,^25^,^26^, and that feral cats prefer to hunt in intensely burnt habitats if available within their home-range^27^. But it seems surprising that cats could be responsible for recent declines, because in the savannas of northern Australia they occur at very low densities (mean 0.18 cats.km^−2^, s.e.m 0.08, in our study area)^28^. They also typically show strong site fidelity and occupy exclusive territories^27^, meaning that individual cats are widely spaced. We lack a mechanistic understanding of how sparse populations of this small predator could be having a substantial impact on prey.

We show that cats make predictable long-distance expeditions to hunt intensively at recent high intensity fires far outside their home ranges, and then repatriate to the original home range. These journeys range up to 30 km, almost ten times the typical home-range diameter. We analyse the factors that influence cats’ decisions to travel, durations of residence at the destination, and finally, the movement rules followed by journeying cats. We argue that the behavioural capacity of feral cats to undertake long-distance excursions to exploit transient hunting opportunities results in significantly higher total predator pressure on prey, and helps to explain how low-density cat populations could have large impacts on small-mammal abundance at landscape scales. This will also have profound implications for global trends of intensified fire regimes^29^.

## Results

**Figure 1:**
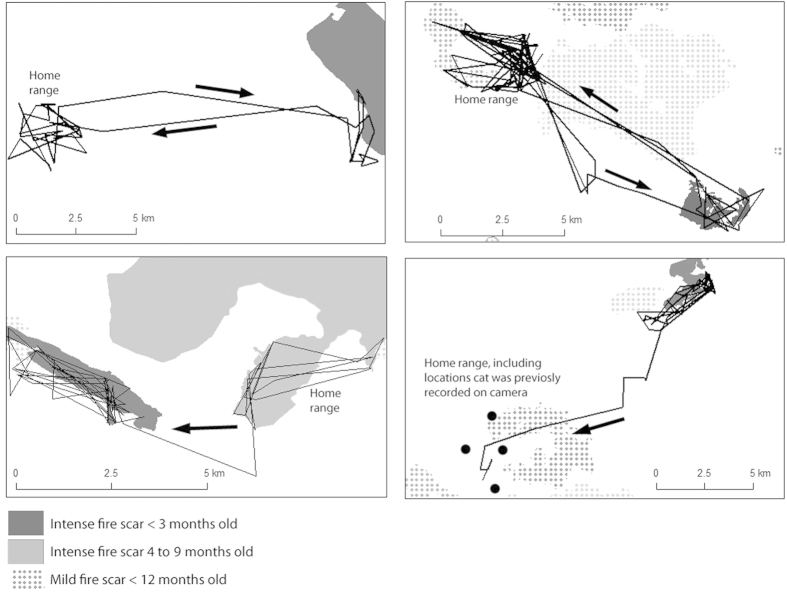
Examples of four of the eight extra-territorial travels to intense fire scars by cats. We created maps in ArcMap 10.1 (www.esri.com), and refined symbology in Photoshop Elements 8.0 (www.adobe.com/au/products/photoshop.html).

We studied movements of 32 cats (25 males and seven females) using GPS tracking between 2010 and 2013 in the Kimberley region of north-western Australia, acquiring a total of 121 cat-months of movement data. Movement paths of cats were profiled into three types: (i) within home-range movements, (ii) long-range journeys, and (iii) area-restricted movements at a destination. We identified eleven clear instances of long-distance journeys, ranging from two to more than fifty days between departure and return (Table 1). Cats journeyed up to 30 km away from their home ranges, but all area-restricted movements at distinct destinations were within 12.5 km of the home range. Eight of the eleven expeditions involved arrival at a distinct destination, defined as a site where a pattern of area-restricted search was initiated (see Fig. 1). One cat died at its destination, so we cannot separate this travel from dispersal movements. Another was initially caught and collared at what we believe was its destination, not its home range. Its pelage matched that of a cat from 12 km away, who was known from a series of infrared camera records at that site over two years. It returned to that site after one month of GPS tracking. All journeying cats were adult males ranging in weight from 3.2–5.1 kg.

To examine factors that elicited long-distance extraterritorial journeys, we created 480 discrete-choice models estimating the likelihood of a cat staying within its home range or traveling up to 12.5 km distant from its home range (the maximum distance of any destination from a home range) for any month, in relation to fire and environmental variables. The most parsimonious model demonstrated that cats had a very strong fidelity to their home range, but that selection for recent intense fire-scars within 12.5 km was even stronger. It had an Akaike weight = 0.58 and AICc of 12.90, compared to only other model in choice set with an Akaike weight = 0.21 and AICc of 13.59 for the next highest model. Both variables had large effect sizes, with a home range coefficient of 11.3 (z = 2.75, P = 0.01) and an intense fire scar coefficient of 21.04 (z = 2.54, P = 0.011). Other habitat variables (e.g. mild fire scars, riparian habitats) did not improve model performance. For example, no cat travelled to a mild fire scar, despite 22 cats having the option to do so. The model itself was significant (Wald test = 7.97, df = 2, P = 0.02), and predicted cat selection in all but one of the 126 choice sets (the exception being the cat that travelled to the edge of a fire scar, top right in Fig. 1). Home ranges at destinations contained far more scars of intense fire than the surrounding landscape: between 43% and 96% cover by fire scars compared to 2% to 27% in the surrounding 12.5 km buffer. Of 12 cats that were monitored during periods when intense fire-scars covered more than 1% of the area within a 12.5 km radius, eight set out on journeys to visit a fire scar. Two of the non-travellers were female that appeared, on the basis of infrared camera images, to be lactating at the time. Therefore, 80% of adult male cats that could have journeyed to a fire did so.

**Figure 2:**
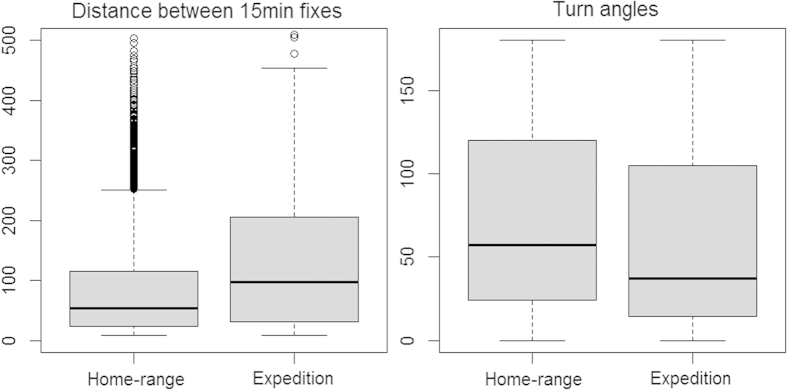
Comparison of movement parameters between 15 minute fixes within cats’ home-ranges, and during expeditions to and from an intense fire scars. Graphs were generated in R 3.1.3 (https://www.r-project.org).

We compared movement parameters of expeditions against within-territory movements, and found cats were travelling faster (linear mixed effects, DF = 8865, t = 13.12, P < 0.01) and more likely to be heading forwards (linear mixed effects, DF = 8865, t = 9.72, P < 0.01) (see Fig. 2). To determine whether cats’ journeys were purposefully directed towards intense fire scars, we compared each actual journey with 100 correlated random walk simulations using the same number of segments, along with the same turn-angles and segment lengths; yet with the sequence of segments randomised. Compared to actual journeys, the simulated random walks almost never reached an intense fire scar (2% of walks reached the fire, F = 2781.3, P < 0.001), had a far higher tortuosity (Fractal D of 0.7 vs 0.22, F_1,8_ = 52.5, P < 0.001), and were less likely to begin in the direction of a fire scar (F_1,8_ = 27.5, P < 0.001). There was no dominant bearing of journeys (Table 1).

**Figure 3:**
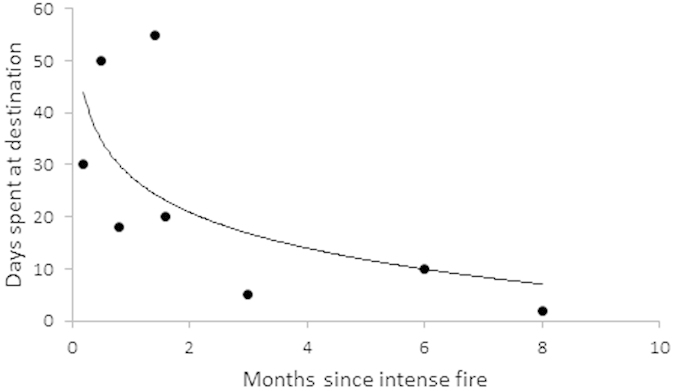
For cats expeditions to an intense fire scar, the duration of their visit in days is compared against the months since an intense fire at the destination. Graph was generated in Microsoft Excel 14.0.6112 (https://products.office.com/en-au/excel).

Once cats reached their destinations, their duration of stay was on average 15 days, but increased with recency of the fire (Fig. 3). From a choice set of three, the linear model with the lowest AICc (26.09 vs 28.42) and highest Akaike weight (0.58, vs 0.31 and 0.11) contained the single variable time since intense fire log transformed (value = −0.34, t = −3.8, P = 0.01); the model was significant (F = 14.15, df = 6, P = 0.01; R^2^ = 0.7).

## Discussion

This is the first report of long-distance extraterritorial expeditions by feral cats to short term and unpredictable pulses of prey availability. Male feral cats made fast, straight, directed movements over long distances to intense fire scars, even though they otherwise held exclusive home ranges^28^,^30^. Although the number of recorded journeys was small (11), the consistent destinations and strongly directed character of the movements make these data compelling. Even though all travelling cats were male, we do not believe these journeys had the objective of finding mates. The area-restricted searches that were initiated at destinations were at too large a spatial and temporal scale to be consistent with mating. Also, cats in the study area select for such intense fire scars within their home range^27^, and the long-distance movements reported here appear to be an extension of that behaviour. While 80% of the male cats exposed to an intense fire within 12.5 km of their home range journeyed to it, none of the 22 cats with a mild fire within 12.5 km journeyed, probably because such fires leave pockets of unburnt grasses that can provide refuge for prey and make hunting less profitable for cats^31^. We suggest that cats make these journeys to intense fire scars to take advantage of short pulses of high prey availability, where no such refuges remain^32^. Prey of feral cats (e.g. rodents) benefit from grass cover for protection from predators^33^,^34^,^35^, and the complete loss of such would create ideal hunting areas for predators.

These long-distance journeys by cats could create sudden dramatic increases in density of cats at fire scars, which could in turn have an aggregate effect of driving down the density of small mammals over whole landscapes. An influx of predators to a fire scar would result in temporarily elevated rates of predation, and potentially even local extirpation. If such fires recurred with sufficiently high frequency, and in a pattern that left few areas unburnt, it is possible that elevated predation due to fire could reduce small mammals and prevent recovery over large areas. This could explain how a relatively low population density of cats could have a major impact at a landscape scale, if fire regimes are inappropriate^36^,^37^.

This study demonstrates spatial awareness in feral cats beyond the areas with which they are likely to be familiar from routine home-range movements. Although cats could have visited these destinations before we placed GPS collars on them, they could not have known there would be a pulse of prey availability after an intense fire from memory of prior visits. Possibly, they could have detected fire scars by a cue of either the smell of smoke, smell of ash, observing the glow of fire from a distance, or observing movements of other individuals or species. The smell of smoke or fire glow is unlikely to have been used as a direct beacon for navigation, as there was a time lag between the occurrence of fire and expeditions of five days or more, over which time such cues would have lapsed. Yet it is possible the cats retained the memory of the direction of smoke or fire glow for days and up to several months before they embarked on their expeditions. While the smell of ash might still be present for many months post-fire, this is unlikely to be the primary cue, as such smells would also linger on all the mild fire scars that cats did not move to. Cats might have learnt navigation cues from other individuals or species (e.g. birds of prey), however, we cannot provide any supporting or detracting evidence for this. Regardless of the cues they use, the accuracy of their expeditions to and from these intense fire scars demonstrates exceptionally large spatial scales for their mental maps.

Two of the greatest threats to wildlife globally are intensifying fire regimes and predation by invasive predators, and our documentation of interaction between these factors has major implications for conservation^38^. In general, fire regimes are intensifying due to anthropogenic land-use change^39^, invasions of exotic flammable grasses^40^,^41^, and global warming^42^,^43^. The latter would have the most profound long-term impact, as rainfall variability and hence extreme fire conditions will increase in all modelled scenarios^29^. For wildlife, this means that not only will animals have to withstand the direct effects of these fires^34^, but may also be subject to increased predation. Our new understanding of the behaviour of feral cats provides even stronger grounds for reducing the frequency and extent of intense fires in the northern savannas of Australia wherever possible.

## Methods

### Study area

Our study area was in north-western Australia, at a pastoral station and two wildlife sanctuaries managed by the Australian Wildlife Conservancy in the central Kimberley (17°S, 126°E). Habitats are savanna woodlands with a perennial grass layer, dissected by riparian vegetation along the edges of creeks. Fire is managed on all three properties to promote biodiversity values. This involves reducing the incidence of extensive, intense fires in the late dry season by lighting strategic prescribed fires with incendiaries in the early dry season when fires are small-scale and mild.

### GPS tracking of cats

We studied movements of 32 cats that were captured and had GPS telemetry collars (Telemetry Solutions, Quantum 4000 enhanced) attached between 2010 and 2013. Cats weighing between 2.0 and 3.3 kg were fitted with a 70-g collar (25 × 15 × 50 mm) , and those more than 3.3 kg were fitted with a 100-g collar (20 × 20 × 50 mm) ensuring all collars were less than 3% of body weight. Cats were caught using either large wire cage traps, leg-hold traps (soft-jaw, size #1.5) or by spotlighting and netting with the assistance of dogs trained to locate and corner cats. Collars were set to record one fix per day at 20:00 pm WST, with bouts of fifteen minute fixes each of two days duration. The single fix per day was used for home range and destination area calculations, while the fifteen-minute fixes were used to measure the properties of the transit passage. The majority (73%) of transit passages were captured in the fifteen-minute fixes.

### Characteristics of movements by cats

To investigate expeditions by cats outside their home-ranges, we created definitions and ‘triggers’ for switching between three movement states: within home-range movements, transit, and area-restricted movements at destinations. To define the distinction between within home-range movements and transit, for every fix we determined the distance from the cumulative mean home-range centroid, kept a running standard deviation, and deemed any fix greater than two standard deviations away from this range to represent an abrupt move outside a home-range boundary. To differentiate transit from an area-restricted search, we used first-passage time^44^. For all fixes within a sequence outside a home-range, we calculated the first passage time for circles of 100 m increments, the log-variance for each increment, selected the circle size with the greatest log-variance, and used that size circle to determine area-restricted movements outside a home range. In all instances, home-range movements, transit, and area-restricted movements at destinations were unambiguous (see Fig. 1 for examples).

Once travels had been defined, we used discrete-choice modelling to identify determinants of the cats’ decisions to travel. Every complete month of active GPS fixes was analysed as a ‘choice’ of whether to stay or travel, and where to choose as the destination. The choice set was defined as the home range, and five randomly generated possible destinations outside the home range. As we had too few journeys to generate a probability distribution, these ‘available’ possible destinations were defined using these five randomly placed sites the size of the average area-restricted destination within a 12.5 km buffer around the home range (the longest journey of a cat during this study). For cats that travelled to a destination in any month, the destination was added to the choice set and marked as chosen, even if the cat was not there for the whole month. The areas and shapes of home ranges and destinations were defined from a kernel density estimate of all suitable fixes using smooth cross validation, around a 95% isotope.

To determine predictors of travel in a spatially and temporally dynamic landscape, we measured landscape features relevant to cats for each month. Fire extents were mapped using monthly Landsat 7 remote-sensing imagery available from the US Geological Survey (2011–2013). For each burnt area, we assigned the date of burn, the intensity (intense = 100% tree scorch and no ground cover remaining unburnt, or mild = all other fires). Fire was classed by intensity in binary variables at one, three, six, nine and twelve months since fire, relative to the date of each GPS fix. Regions were divided into relative prey abundance based on average number of small mammals caught per year per area, from an annual dataset (Australian Wildlife Conservancy, unpublished data). We also calculated percent cover of riparian habitats, known to be important to cats^27^. The percent of each habitat feature was measured for the home range and five possible destinations for each month of cat movement data. Standard case-control logistic regression models were implemented in R v. 8^45^ using the ‘survival’ library^46^, with a separate model for every combination of variables. These models were compared within an information theory framework to select the most parsimonious model.

We compared movement parameters (distances between successive fixes and turn-angles) of expeditions against within-territory movements using linear mixed effects models, with individual cats as an error term. Both variables were log-transformed to meet assumptions of normality. Only the movements of cats that made expeditions were considered. To determine whether journeys consisted of directed movement that efficiently reached a destination, we compared each actual journey against 100 simulations of biased random walks. These walks were based on the 15-minute segments. We detected significant correlation in distances travelled between successive fixes using Jlung-box tests^47^, where cats were more likely to move greater distances if previous step lengths were longer and turn angles were shorter. This bias was incorporated into the selection of random step-lengths. Each simulation used the same number of segments as the corresponding journey, and step-lengths and turn angles were drawn from the same probability distributions as journeys. We counted how many of the 100 simulations reached the destination, and compared this with observed values using an analysis of variance test. To determine whether routes taken were more direct or tortuous than expected, we calculated the Fractal D^48^ for each journey; direct distance between start and finish divided by the distance of route travelled. We also compared the bearing of the first three segments of travel against the bearing of destination.

We measured length of time at destination against the months since the intense fire, season, prey density at destination. Linear models were fitted to every combination of the above variables. Time at fire was log-transformed to fulfil assumptions of normality. Models were compared within an information theory framework.

### Ethics statement

All field methods were conducted in accordance to procedures that were approved by both University of Tasmania, and Western Australian Department of Parks and Wildlife Animal Ethics committees.

## Additional Information

**How to cite this article**: McGregor, H. W. *et al*. Extraterritorial hunting expeditions to intense fire scars by feral cats. *Sci. Rep*. **6**, 22559; doi: 10.1038/srep22559 (2016).

## Figures and Tables

**Table 1:** Details of the 11 recorded journeys of feral cats.

Cat name	Home range size (ha)	Max distance from home range	Broad bearing of travel	Destination details	Destination size (ha)	Days at destination
Bruce	1104	12 km	East	intense fire scar edge	558	30
Askelladd	891	10 km	North-east	intense fire scar	718	10
	891	30 km	North-east	intense fire scar edge	172	2
Smokey	375	1 km	North-west	intense fire scar	2006	>51
	375	20 km	North-east	none		NA
Storm	445	2 km	South-west	none		NA
Pork noodle	904	5 km	West	intense fire scar	490	35
Mike	971	4 km	South-east	none		NA
	971	11 km	South-east	intense fire scar	726	20
	971	11 km	South-east	intense fire scar	543	5
Jaws	560	8 km	South-west	intense fire scar	826	>42
